# Glycyrrhizae Radix et Rhizoma Processed by Sulfur Fumigation Damaged the Chemical Profile Accompanied by Immunosuppression and Liver Injury

**DOI:** 10.1155/2020/5439853

**Published:** 2020-02-06

**Authors:** Jun Jiang, Shichang Xiao, Shu Yan, Jianpeng Xiao, Ximing Xu

**Affiliations:** ^1^School of Pharmacy, Jiangsu University, 301^#^ Xuefu Road, Zhenjiang 212013, Jiangsu Province, China; ^2^ADR Monitoring Center, Zhenjiang Food and Drug Supervision and Inspection Center, 62^#^ Nanxu Road, Zhenjiang 212000, Jiangsu Province, China

## Abstract

Glycyrrhizae Radix et Rhizoma (GRER) has been used as a medicinal plant and dietary supplements for its beneficial effect in immunomodulatory effects. Sulfur fumigation (SF) processing was widely used in the storage and maintenance of Chinese medicine because of its convenience and cheapness. However, the disadvantage of SF has been reported, but the systematic study of SF on GRER was deficient. In this paper, the active ingredients, sulfur-fumigated products, immunomodulatory effect, and liver injury of SF-GRER were studied. After SF, the liquiritin decreased from 4.49 ± 0.03 mg/g to 3.94 ± 0.08 mg/g (*P* < 0.01). Compared with the NSF-GRER group, the SF-GRER group showed a decreased immunoregulation in the thymus index, spleen index, and serum IL-6 and SOD levels (*P* < 0.05). After 2 weeks of continuous intragastric administration of SF-GRER in healthy mice, the level of serum aspartate aminotransferase (AST) significantly increased (*P* < 0.05) and the area of liver lesion significantly increased compared with the NSF-GRER (*P* < 0.05) group. The sulfonated products (*m*/*z*, 631.13) corresponding to liquiritin apioside (*m*/*z*, 551.17) and isoliquiritin apioside (*m*/*z*, 551.17) were screened out in SF-GRER by using UPLC-Orbitrap-MS. The sulfonated products provided in this paper were discovered for the first time and could be powerfully applied for the identification of SF-GRER. SF destroyed the chemical composition of GRER, inhibited immunoregulation, and induced liver injury. The feasibility of this processing method needs to be reconsidered.

## 1. Introduction

Glycyrrhizae Radix et Rhizoma (GRER) has been used as a medicinal plant and dietary supplements since ancient times in China, Japan, Italy, United States, and other different countries and regions [1–4]. Modern research studies showed that GRER had extensive pharmacological effects in antispasmodic, antidiabetic, antiosteoporosis, antidepressive, antitussive and expectorant, hepatoprotective, and memory-enhancing [5–11]. The major bioactive components of GRER were saponins, flavonoid glycosides, and free phenolic compounds [12]. Approximately 250 compounds have been reported from GRER, and more than 151 compounds in GRER have been determined up to present [13]. In the clinical practice of traditional Chinese medicine, there was a saying of “Ten prescriptions and Nine GRER.” Therefore, the clinical demand of GRER is very large.

In recent years, sulfur fumigation (SF) has been used to replace traditional sun-drying for various Chinese herbal medicines to prevent pest infestation, mold, browning, and bacterial contamination in a cheap and convenient manner. The raw materials were usually stacked together, and a pot of burning sulfur was placed at the bottom and fumigated in a closed space for 12–24 hours. There have been many reports of sulfur-fumigated traditional Chinese medicine, including ginseng [14], Angelicae sinensis Radix [15], Chrysanthemum morifolium flowers [16], Ophiopogonis Radix [17], Atractylodes macrocephala Koidz. [18], Fritillaria thunbergii Miq. [19], Radix Paeoniae Alba [20], Smilacis Glabrae Rhizoma [21], Gastrodia Rhizoma [22], and lots of others. Numerous studies have shown that SF-induced dramatic changes in chemical profiles which makes the safety and effectiveness of sulfur-fumigated traditional Chinese medicine have hidden dangers [23]. Therefore, the investigations of SF on chemical profiles, bioactivities, and toxicity of Chinese medicine are urgently needed for reevaluating this potentially harmful processing method. However, the effects of SF on active components, immunomodulation, and liver function of GRER and how to identify sulfur-fumigated GRER are still deficient.

In the present study, firstly, the content changes of main components in GRER before and after SF were detected by HPLC. Secondly, the immunosuppressive mice model and healthy mice model were, respectively, applied to investigate the effects of SF on immunoregulation and liver injury of GRER. Finally, liquid chromatography coupled with linear ion trap hybrid orbitrap mass spectrometry (UPLC-Orbitrap-MS) method was applied to screen the characteristic products in sulfur-fumigated GRER.

## 2. Materials and Methods

### 2.1. Ethics Statement

All animal experiments strictly comply with the Guidelines for Animal Experimentation of Jiangsu University (Zhenjiang, China), and the protocol was approved by the Animal Ethics Committee of this institution.

### 2.2. Materials and Chemicals

GRER was purchased from Gansu Province (Lanzhou Minxian Special Products Co., Ltd.) and identified as the dried roots and rhizomes of *Glycyrrhiza uralensis* Fisch. by Professor Wu Chengying in Jiangsu Academy of Traditional Chinese Medicine. Liquiritin (no. K186728, purity > 99.0%) was purchased from Xi'an Kaili Bioengineering Co., Ltd (Xi'an, China). Sublimated sulfur was purchased from Chemical Reagents Co., Ltd. of China Pharmaceutical Group (Shanghai, China). The chromatographic distilled water was made in our laboratory. Chromatographic pure acetonitrile and methanol were purchased from Shanghai Xingke High Purity Solvent Co., Ltd (Shanghai, China). Analytical pure sodium chloride, cyclophosphamide, formaldehyde, and sodium heparin were purchased from Titan Technology Co., Ltd. (Shanghai, China).

### 2.3. Preparation of Sulfur-Fumigated and Nonsulfur-Fumigated Samples

Sulfur-fumigated GRER (SF-GRER) was prepared according to the method used in literature [24]. The appropriate amount of nonsulfur-fumigated GRER (NSF-GRER) and SF-GRER was weighed and extracted twice with 10 times of water. Combined with the two extracts and concentrating to 100 mL under vacuum, the freeze-dried powder was obtained. For the accuracy of the results, 5 batches of SF-GRER and NSF-GRER were prepared in parallel. 0.2 g of freeze-dried powder of NSF-GRER and SF-GRER were separately weighed in a conical bottle, and 50 mL methanol was added immediately. After 30 min of ultrasound-assisted extraction, the membrane-crossed (0.22 *μ*m) extracting solution was used for HPLC-DAD and Orbitrap-MS analysis.

### 2.4. Animals and Administration

Male KM mice (20 ± 2 g) were supplied by Laboratory Animal Center of Jiangsu University. All animals were housed under 20–25°C with a relative humidity of 40–70%. All the experimental protocols were approved by the Animal Ethics Committee of Jiangsu University. Animals were divided into two parts. Part I includes mice divided into 6 groups (*n* = 10), and they were normal control group (CON, normal saline, i.g), model group MOD, after continuous intraperitoneal injection of cyclophosphamide (150 mg/kg) for 3 days, SF-GRER low-dose group (SF-L, 2 g/kg/day, i.g), SF-GRER high-dose group (SF-H, 4 g/kg/day, i.g), NSF-GRER low-dose group (NSF-L, 2 g/kg/day, i.g), and NSF-GRER high-dose group (NSF-H, 4 g/kg/day, i.g). Part II includes healthy mice separately gavaged in NSF-GRER or SF-GRER at high or low doses (the same dose as part I, *n* = 10) for 14 consecutive days to investigate the effects of SF-GRER on liver function.

### 2.5. Collection and Treatment of Mice Samples

The body weight of mice in each group of part I was recorded daily. After 10 days of continuous administration, the whole blood of mice in each group was obtained by eyeball extraction and the serum was separated by centrifugation. Serum SOD and IL-6, thymus index, and spleen index of every group in part I were detected, respectively. For part II, after 14 days of continuous administration, the serum AST was detected and their liver were obtained and fixed by 10% formalin solution. The liver injury was assessed by HE staining and percentage of lesions.

### 2.6. Sample Analysis

**Figure 1:**
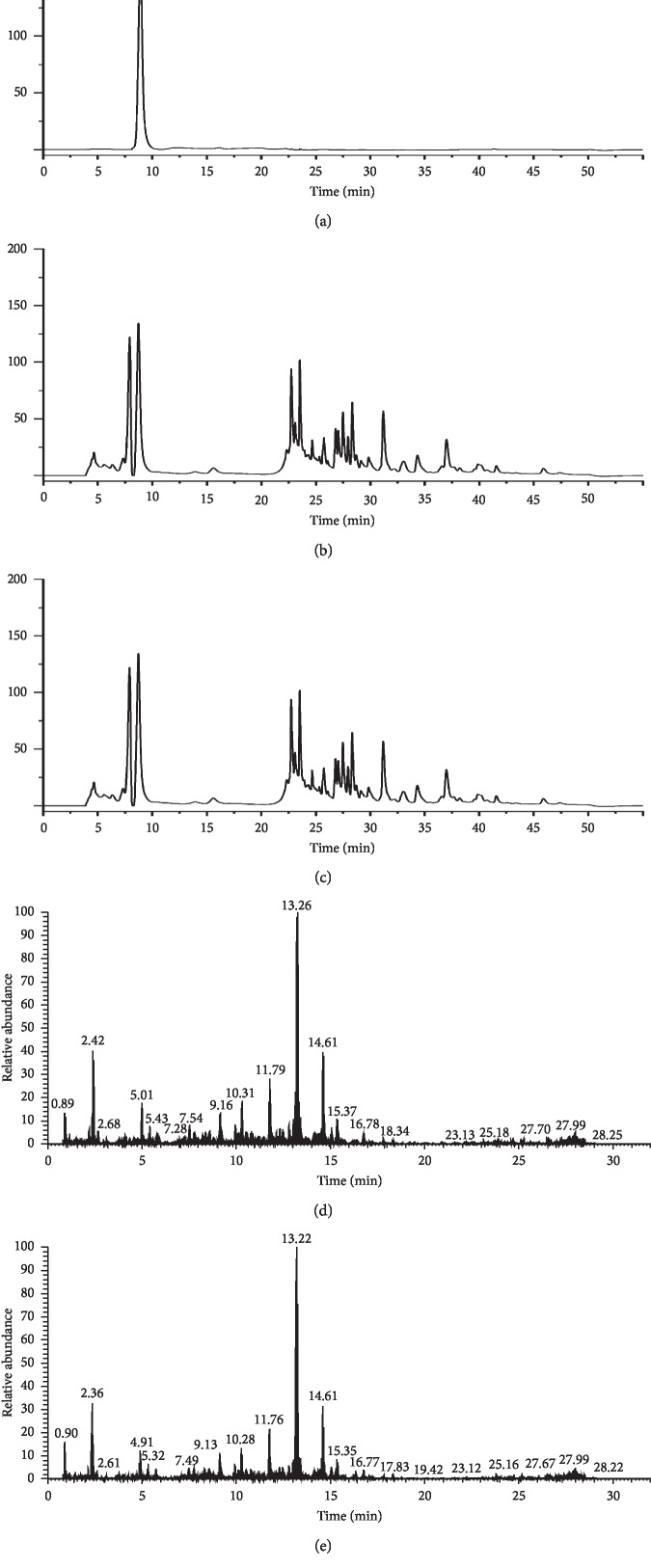
HPLC-DAD and UPLC-Orbitrap-MS chromatogram: (a) chromatography of liquiritin standard solution; (b) chromatography of freeze-dried powder of NSF-GRER extract; (c) chromatography of freeze-dried powder of SF-GRER extract; (d) total ion chromatography of NSF-GRER; (e) total ion chromatography of SF-GRER.

The HPLC coupled with DAD and LCsolution (LC-AT_SR,_ Shimadzu, Japan) was used for the content determination of liquiritin. The chromatographic separation was operated with an Agilent Zorbax SB-C_18_ column (4.6 mm × 250 mm, 5 *μ*m) under column temperature 40°C, detection wavelength 275 nm, flow rate 1.0 mL/min, and injection volume 20 *μ*L. The mobile phase was acetonitrile (A) and water (B) under a gradient elution (0–15 min, 18% A; 15–20 min, 18–60% A; 20–30 min, 60% A; 30–40 min, 60–18% A; and 40–42 min, 18% A; Figures 1(a) and 1(b)). UPLC-Orbitrap-MS was equipped with an electrospray ionization source under positive mode. Chromatographic separation was performed using a Dionex U3000 UPLC system with a Phenomenex Kinetex C_18_ column (2.1 mm × 100 mm, 2.6 *μ*m). The column temperature was set at 30°C. The mobile phase consisted of solvent A (acetonitrile) and solvent B (0.1% formic acid in water) with gradient program: 0 to 5 min, 5% A; 5 to 15 min, 5–35% A; 15 to 20 min, 35–85% A; 20 to 28 min, 85–5% A; and 28 to 30 min, 5% A (Figures 1(c) and 1(d)). The flow rate was 0.25 mL/min, and the sampling volume was 5 *μ*L. For the MS conditions, the spray voltage was 3.5 kV, the heated capillary temperature was 300°C, the ESI probe temperature was 350°C, the flows of sheath gas and auxiliary gas were 40 units and 15 units, respectively, the scan range was 500–1800 *m*/*z* with the Orbitrap analyzer, and target ions selected for fragmentation were obtained by dynamic exclusion for 20 s. All the data analysis was performed using Xcalibur 2.2 SP1 software (Thermo Fisher Scientific, Inc., Bremen, Germany).

### 2.7. Biochemical Indices and Pathological Evaluation

The thymus and spleen index were calculated as follows: spleen (thymus) index = the spleen (thymus) mass (mg)/the body weight (g). Blood samples were collected from eyeballs at intervals of one hour after the last administration. The blood samples were placed in a 2 mL centrifugal tube containing 20 *μ*L of 5% heparin sodium. The supernatant was centrifuged at 3700 rpm for 10 min and frozen (−80°C) in the centrifugal tube. Serum SOD, IL-6, and AST were determined by ELISA [25]. HE staining sectioning (2 *μ*m) was read under optical microscope (OLYMPUS BX41, Japan) [26]. The main examination of the liver was (1) whether there is hepatocyte degeneration (steatosis and edema), (2) whether there is hepatocyte degeneration and necrosis, (3) whether there is inflammatory cell infiltration in the liver lobule, (4) whether there is inflammation or fibrous tissue proliferation in the portal area, and (5) whether there is atrophy of hepatocyte and expansion of hepatic sinuses. Five visual fields were selected for each slice, and then the average percentage of lesion in different groups was calculated.

### 2.8. Statistical Analysis of Data

Statistical analyses were conducted using one-way ANOVA (GraphPad Prism 5.0) followed by the Tukey test for comparing all pairs of columns. Data were presented as mean value ± SD. The *P* value <0.05 was considered as statistically significant.

## 3. Results

### 3.1. Methodological Validation

The standard solution was determined according to the chromatographic conditions under item “*HPLC-DAD analysis*.” The peak area of liquiritin (*Y*) was linearly regressed by sample concentration (*X*). The results showed that the linear relationship of liquiritin was fine in the range of 20–200 *μ*g/mL. The regression equation was *Y* = 42438*X* − 232006, *r*^2^ = 0.9998. The RSD of intra- and interday precision was 0.32% and 0.51%, indicative of a high precision. The RSD of repeatability in SF-GRER and NSF-GRER was 0.67% and 1.98%, respectively. The detection limit (LOD) and quantitative limit (LOQ) of liquiritin was 0.06 *μ*g/mL and 0.21 *μ*g/mL, respectively. The recovery rates of liquiritin in SF-GRER and NSF-GRER were 100.37% and 100.27%, respectively (Table 1). The method had superior stability, reproducibility, and recovery which ensured the accuracy of the quantitative results.

### 3.2. Liquiritin before and after Sulfur Fumigation

The liquiritin in NSF-GRER was 4.49 ± 0.03 mg/g (0.449%). Compared with NSF-GRER, the liquiritin in SF-GRER decreased significantly to 3.94 ± 0.08 mg/g (0.394%, *P* < 0.01). The liquiritin in GRER before and after sulfur fumigation was shown in Table 2. Our results showed that liquiritin was destroyed or transformed into other structures during sulfur fumigation.

### 3.3. Immunomodulatory Effects before and after Sulfur Fumigation

**Figure 2:**
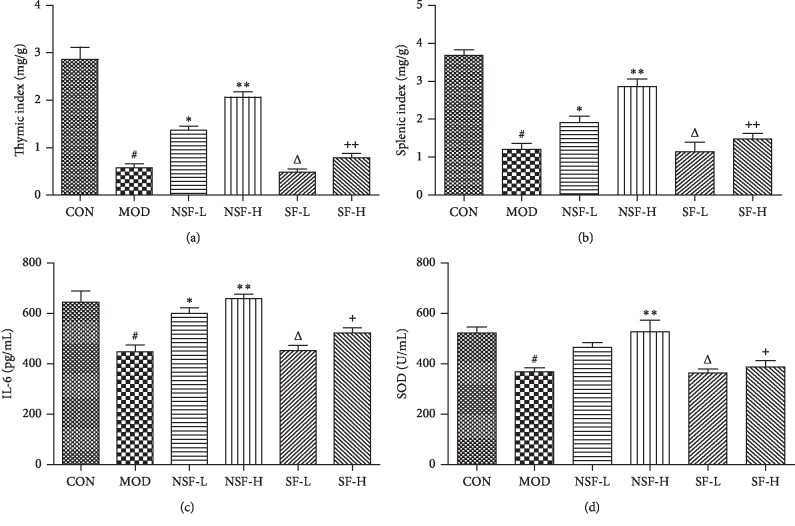
Immunoregulation of GRER before and after sulfur fumigation ($\bar{x}$ ± SD, *n* = 10). ^#^Compared with CON, *P* < 0.01; ^*∗*^compared with MOD, *P* < 0.05; ^*∗∗*^compared with MOD, *P* < 0.01; ^∆^compared with NSF-L, *P* < 0.05; ^+^compared with NSF-H, *P* < 0.05; ^++^compared with NSF-H, *P* < 0.01.

Compared with the healthy mice, the thymus index (0.58 ± 0.15 mg/g), spleen index (1.20 ± 0.36 mg/g), serum IL-6 (448.00 ± 54.40 pg/mL), and SOD (367.50 ± 33.67 U/mL) levels of the model group were significantly decreased by cyclophosphamide (*P* < 0.01). Under the intervention of NSF-GRER (low and high dose), the immunosuppression induced by cyclophosphamide could be reversed and the thymus index (1.37 ± 0.16 and 2.06 ± 0.19 mg/g), spleen index (1.91 ± 0.39 and 2.86 ± 0.35 mg/g), serum IL-6 (600.11 ± 46.44 and 658.77 ± 35.99 pg/mL), and SOD levels (465.00 ± 37.97 and 527.50 ± 92.11 U/mL) were significantly increased (*P* < 0.05). However, with the intervention of SF-GRER, the indicators showed an upward trend but no significant difference. It is worth noting that, compared with the NSF-GRER group, the SF-GRER group showed a significant decrease in immunoregulation (*P* < 0.05). Detailed results are shown in Figure 2.

### 3.4. Effects on Liver Function

**Figure 3:**
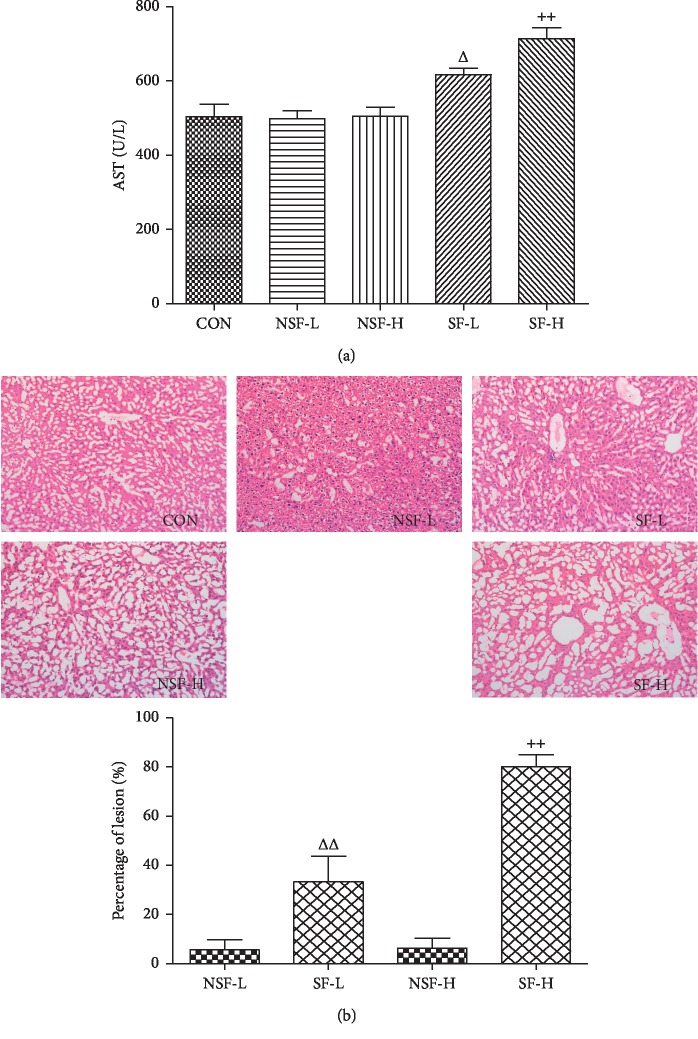
Effects of SF-GRER on liver function in healthy mice ($\bar{x}$ ± SD, *n* = 10): (a) effect of sulfur-fumigated GRER on the serum AST level in healthy mice; (b) representative photomicrographs (×100) and lesion assessment of livers after prevention of NSF-GRER and SF-GRER. ^∆^compared with NSF-L, *P* < 0.05; ^∆∆^compared with NSF-L, *P* < 0.01; ^++^compared with NSF-H, *P* < 0.01.

After 2 weeks of continuous intragastric administration of SF-GRER and NSF-GRER in healthy mice, the level of AST increased significantly in SF-GRER mice (*P* < 0.05, Figure 3(a)). Therefore, we suspected that SF-GRER may induce liver damage in healthy mice [27, 28]. Subsequent pathological studies showed that SF-GRER significantly increased the area of liver lesion compared with the same dose of NSF-GRER (*P* < 0.05, Figure 3(b)).

### 3.5. Screening of Sulfur-Fumigated Characteristic Products

**Figure 4:**
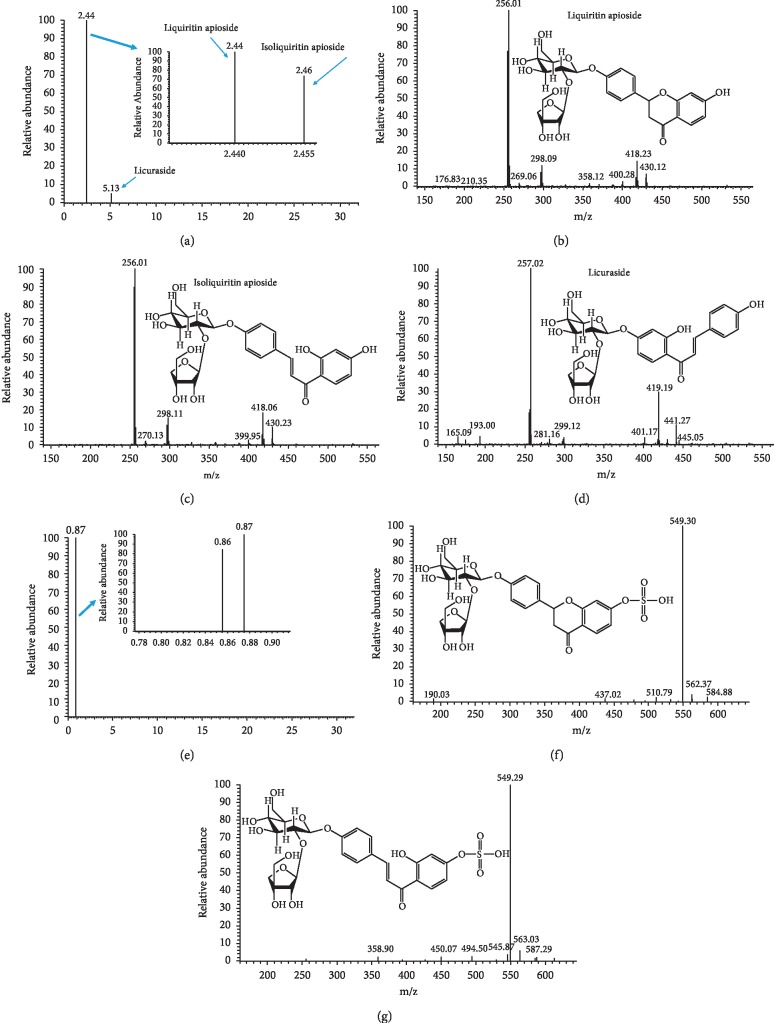
UPLC-Orbitrap-MS spectrum of characteristic products in SF-GRER. (a–d) MS spectrum of liquiritin apioside, isoliquiritin apioside, and licuraside in NSF-GRER. (e–g) MS spectrum of liquiritin apioside sulfonate and isoliquiritin apioside sulfonate in SF-GRER.

**Figure 5:**
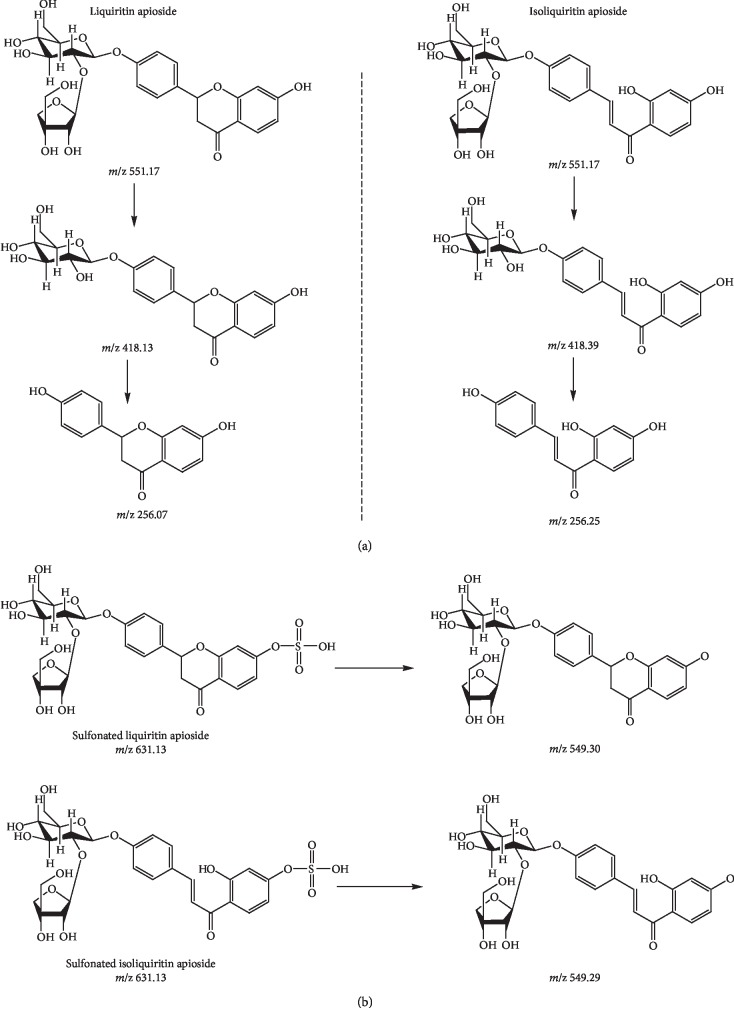
Pyrolysis process of characteristic products in SF-GRER by using UPLC-Orbitrap-MS: (a) pyrolysis of liquiritin apioside and isoliquiritin apioside; (b) pyrolysis of liquiritin apioside sulfonate and isoliquiritin apioside sulfonate.

UPLC-Orbitrap-MS has been widely used in the identification of characteristic components of traditional Chinese medicine [29, 30]. According to related literature [2, 4, 13, 31–34] and the transformation rule before and after SF, the molecular weight ranging from 500 to 1800 (*m*/*z*) was screened one by one under the UPLC-Orbitrap-MS system. Fortunately, liquiritin apioside (LA, *m*/*z*, 551.17) and isoliquiritin apioside (ILA, *m*/*z*, 551.17) not only had been found in NSF-GRER, their corresponding sulfonated products (*m*/*z*, 631.13) also had been screened out in SF-GRER (Table 3 and Figure 4). It is interesting that LA and ILA had same fragmentation ions (*m*/*z*, 256.01 and 418.23) and extremely close retention time (2.44 and 2.46 min) because they were isomers. Sulfonation increased the polarity of LA and ILA, so retention time of their sulfonates was advanced to 0.86 and 0.87 min, respectively. The pyrolysis process of LA and ILA in mass spectrometry was first to remove *β*-apiose (*m*/*z*, 418.13 or 418.39) and then *β*-D-glucose (*m*/*z*, 256.07 or 256.25, Figure 5(a)). The pyrolysis process of LA-SO_3_ and ILA-SO_3_ in mass spectrometry was to remove sulfonic acid groups (*m*/*z*, 549.30 or 549.29, Figure 5(b)). There were no other sulfur-fumigated characteristic products found in such a large amount of screening work.

## 4. Discussion

The advantages to Q-TOF tandem analyzers are their higher mass accuracy and faster scan speeds and duty cycles. However, Orbitrap analyzers do not use magnetic fields to operate, and therefore, cryogenic refrigerants such as liquid helium are not necessary and operating costs are kept low. Furthermore, one of the major advantages of the orbitrap analyzer is its high resolving power [35]. Considering these factors, Orbitrap was applied in this paper.

Liquiritin is one of the main active ingredients in GRER which is also an important index for judging the quality of GRER [36]. Pharmacological studies have shown that liquiritin can enhance immune regulation [37]. Therefore, the liquiritin in GRER decreased significantly after sulfur fumigation, which inevitably leads to the decrease of the immune regulation of GRER. Our results also demonstrated that the decrease of liquiritin in GRER after sulfur fumigation was accompanied by a significant decrease in its reversal of immunosuppressive effect induced by cyclophosphamide in mice. Our results confirm that sulfur fumigation damaged the active ingredients in GRER and reduced its pharmacological activity.

More seriously, the level of serum AST and the area of liver lesion increased significantly after continuous administration of sulfur-fumigated GRER in mice. These results suggested that sulfur-fumigated GRER had potential safety hazards, especially increasing the risk of liver injury. Therefore, whether the sulfur fumigation method can be used in GRER is a question worthy of serious consideration. How to effectively identify sulfur-fumigated GRER which has entered the market is also an important technical problem faced by scientific researchers.

In Chinese Pharmacopoeia, the quality control of sulfur-fumigated Chinese medicines is only to require the control of SO_2_ residue less than 400 mg/kg [38], which is an important indicator to test whether the medicine has been fumigated by sulfur or not (NSF-GRER: 43 mg/kg and SF-GRER, 910 mg/kg). However, the residual amount of SO_2_ is extremely unstable, which will be significantly reduced with the extension of storage time, even lost more than 50% in 4 months [39]. In order to identify sulfur-fumigated medicinal materials effectively, we suggested that the detection of characteristic products for sulfur-fumigated medicinal materials should be added to the pharmacopoeia. Excitedly, in this study, we successfully used UPLC-Orbitrap-MS technology to screen the characteristic products (*m*/*z*, 631.13) of sulfur-fumigated GRER, which can be applied for the identification of sulfur-fumigated GRER.

## 5. Conclusions

The processing of sulfur fumigation destroyed the active ingredients and attenuated the immunomodulatory effect of GRER. More importantly, the sulfur-fumigated-GRER induced liver damage. In this study, the characteristic products of SF-GRER were first discovered, which provided powerful technical support for its effective identification.

## Figures and Tables

**Table 1:** Recovery of liquiritin in NSF-GRER and SF-GRER by HPLC.

Samples	Original quantity (mg)	Added	Determined	Recovery	Average (%)	RSD
NSF	0.449	0.400	0.853	101.10	100.27	1.08
	0.459	0.400	0.857	99.55		
	0.447	0.400	0.846	99.83		
	0.455	0.400	0.862	101.70		
	0.453	0.400	0.849	99.15		
SF	0.400	0.400	0.795	98.70	100.37	1.47
	0.402	0.400	0.80465	100.65		
	0.391	0.400	0.801	102.55		
	0.401	0.400	0.798	99.35		
	0.397	0.400	0.799	100.60		

**Table 2:** Liquiritin in GRER before and after sulfur fumigation (*n* = 3).

No.	NSF (mg/g)		SF (mg/g)	
	Content	Average content	Content	Average content
1	4.524	4.49 ± 0.03	3.864	3.94 ± 0.08^##^
2	4.472		3.934	
3	4.446		3.884	
4	4.485		3.947	
5	4.506		4.064	

^##^Compared with NSF, the content decreased significantly (*P* < 0.01).

**Table 3:** UPLC-Orbitrap-MS parameters of characteristic products in SF-GRER.

Name	Retention time (min)	Formula	Precursor ion (*m*/*z*)	Mass fragments (*m*/*z*)
Liquiritin apioside	2.44	C_26_H_30_O_13_	551.17	256.01
				254.94
				418.23
Isoliquiritin apioside	2.46	C_26_H_30_O_13_	551.17	256.01
				418.23
Licuraside	5.13	C_26_H_30_O_13_	551.17	257.02
				419.19
				441.27
Liquiritin apioside sulfonate	0.86	C_26_H_30_O_16_S	631.13	549.30
Isoliquiritin apioside sulfonate	0.87	C_26_H_30_O_16_S	631.13	549.29

## Data Availability

The data used to support the findings of this study are available from the corresponding author upon request.
